# Invasion Expansion: Time since introduction best predicts global ranges of marine invaders

**DOI:** 10.1038/srep12436

**Published:** 2015-07-31

**Authors:** James E. Byers, Rachel S. Smith, James M. Pringle, Graeme F. Clark, Paul E. Gribben, Chad L. Hewitt, Graeme J. Inglis, Emma L. Johnston, Gregory M. Ruiz, John J. Stachowicz, Melanie J. Bishop

**Affiliations:** 1 Odum School of Ecology, University of Georgia, Athens, GA 30602 USA; 2 142 Morse Hall, Ocean Process Analysis Laboratory, 8 College Rd, UNH, Durham NH 03857 USA; 3 Evolution & Ecology Research Centre, School of Biological, Earth, and Environmental Sciences, University of New South Wales, Sydney 2052 NSW Australia; 4 Centre of Marine Bio-innovation, School of Biological, Earth and Environmental Science, University of New South Wales, Sydney 2052 NSW Australia; 5 School of Science, University of Waikato, Hamilton 3240, New Zealand; 6 National Institute of Water and Atmospheric Research, 10 Kyle Street, Riccarton, Christchurch 8011, New Zealand; 7 Smithsonian Environmental Research Center, 647 Contees Wharf Road, Edgewater, MD 21037 USA; 8 Department of Evolution and Ecology, University of California, Davis, CA 95616 USA; 9 Department of Biological Sciences, Macquarie University, North Ryde, NSW 2109 Australia

## Abstract

Strategies for managing biological invasions are often based on the premise that characteristics of invading species and the invaded environment are key predictors of the invader’s distribution. Yet, for either biological traits or environmental characteristics to explain distribution, adequate time must have elapsed for species to spread to all potential habitats. We compiled and analyzed a database of natural history and ecological traits of 138 coastal marine invertebrate species, the environmental conditions at sites to which they have been introduced, and their date of first introduction. We found that time since introduction explained the largest fraction (20%) of the variability in non-native range size, while traits of the species and environmental variables had significant, but minimal, influence on non-native range size. The positive relationship between time since introduction and range size indicates that non-native marine invertebrate species are not at equilibrium and are still spreading, posing a major challenge for management of coastal ecosystems.

Predicting the impact and spread of biological invasions remains a key challenge for environmental management^1^,^2^. Characteristics of the receiving environment, native biota, and the species themselves have all been implicated as determinants of species establishment, spread, and impact^3^,^4^,^5^,^6^,^7^,^8^. Yet there is little agreement on the relative utility or generality of these predictors, despite the fact that they are used to inform our investments in preventing invasion and mitigating impacts. For example, to minimize impact from non-native species should we prioritize approaches that ban importation of species with certain traits, or guard habitats that seem particularly vulnerable?

In assessing invader impact and its key drivers, a quantifiable metric is required. The range size of a non-native species is among the most relevant because when multiplied by a species’ mean density and per capita effects, it determines the species’ total impact^9^. Range is often the easiest component of impact to measure, probably has the smallest estimation error (on the order of 10%)^9^, and can display over seven orders of magnitude variation among species. Furthermore, within a species’ native geographic distribution, range is responsive to species traits (body size, niche breadth) and environmental conditions^10^. For example, among marine invertebrates, species with more mobile or longer-lived dispersal stages have significantly larger native geographic ranges than species with non-feeding, less mobile larvae (e.g.,^11^,^12^,^13^), and body size is also associated with range size within some taxa^14^,^15^. Such patterns within native ranges form the basis for using these traits to predict the potential impact and spread of invasions.

Although species traits and environmental conditions are sometimes good predictors of the range sizes of native species, they may not work well in predicting the novel ranges of introduced species’ where species have not yet reached geographic equilibrium. If the rate of post-introduction dispersal or population growth is low, time since introduction could have a strong influence on the distribution and abundance of these species^16^,^17^,^18^. Thus, although species’ traits and environmental attributes may ultimately predict *the potential range* occupied by species, non-native species may not have been present long enough to spread and occupy the total potential range, making the influences and relative importance of environmental and species traits harder to discern. As a result, species may appear to have weak habitat and environmental affinities if other processes, such as dispersal limitation, delay the occupation of suitable habitats in a significant portion of the potential range^19^. The confounding effects of time could in part be responsible for discord in the science and management communities about the relative importance of different factors driving invasion success^20^, and thus which management options deserve highest priority.

We investigated the ability of introduced species’ traits, environmental attributes of the recipient environment, and time since first introduction globally to explain the non-native geographic ranges of marine invertebrate species. Vectors typically responsible for introducing marine invertebrate species are ships (ballast water, hull fouling), aquaculture imports (primarily oysters and associated hitchhiking species), and live trade (aquarium, live seafood, bait)^21^. Once in a new range a species can continue to spread secondarily through these same human-mediated vectors, as well as through natural dispersal, including passive transport of dispersive stages in the water column (e.g., larvae) and to a lesser degree through active locomotion (e.g., walking or swimming).

Using the Global Biodiversity Information Facility (GBIF, http://www.gbif.org/), we developed a database of 138 species of coastal marine invertebrates that are non-native in Australia, New Zealand, or the United States—well-studied domains with some of the world’s richest data for marine invasions and environmental variables (Supplementary Table 1). To control for variation in data quality, we included in our database only marine invertebrates non-native to these countries that: 1) are present in benthic habitats; 2) are not restricted to brackish water; 3) are not part of known cryptic species complexes (which can enhance the probability of misidentification); 4) have sufficient life history information to contribute to our analyses; and 5) have known native ranges (i.e., not cryptogenic). For each species we logged all recorded locations in the non-native range, tabulated the length of its non-native distribution separately for each continental coastline, and summed these values across coasts to calculate the total non-native range size to be used as our response variable. From the literature we extracted time since the first record of introduction anywhere in the world and life history attributes, including maximum body size, adult mobility (sessile or mobile), adult habitat (epifaunal or infaunal), and larval development type (planktonic or non-planktonic). These variables were selected because they are easy to measure, have widely available information, and have previously been shown to either directly or indirectly influence dispersal and/or population growth rate. We extracted physical data on annual and seasonal averages and standard deviations in water temperature, salinity, and currents for all the GPS points of occurrence in the non-native range from a 1-degree resolution climatology of oceanographic variables. These physical variables address whether there are certain abiotic/environmental properties more associated with invasion, for example, if warmer or saltier domains are more heavily invaded, i.e. promote broader ranges in invasive species. To aid visualization of our results, for each species we also recorded the latitudinal range it occupied along each coastline.

The sizes of species’ non-native ranges along coastlines varied widely, ranging between 50 km (our minimum resolution) and 23,000 km (Fig. 1). The single most important variable in predicting non-native range size was time since first recorded global introduction. Time explained 20% of variation in range size (Fig. 2), and was present in every top fitting model, hence its relative variable importance (RVI) of 1.0 (Table 1). The best-fitting model also contained maximum body size, habitat type, and mean salinity and explained 29% of variation in range size. Among the top models, several other biological and physical variables, especially mean spring water temperature, contributed slightly to the overall fit (Table 1), but little relative to time since invasion (Supplementary Figure 1).

Time since first global record of introduction is a powerful explanatory variable for predicting the current extent of coastal non-native invertebrate species’ distributions. The strong, positive slope of the relationship, with the absence of an asymptote, suggests that many invaders are not yet at geographic equilibrium, and signals their continuing spread. The slope of the relationship (37.8) indicates that non-native species expand on average ~400 km per decade. Although our results do not expose which mechanistic processes are driving expansion as a function of time, many influential ecological and evolutionary processes that affect spread rates of non-native species are a function of time, such as population growth rates, dispersal rates, competitive exclusion, and selection (e.g.,^22^,^23^). Therefore, time may serve, at least in part, as a useful proxy variable that integrates and subsumes the influences of many processes that are harder to measure directly.

Our study marks the first instance that the influence of time since global introduction has been considered comprehensively as a predictor of spatial extent of marine invasions, and it is striking that effects of time since invasion emerge despite considering taxa from 10 phyla, spanning diverse life histories and ecological characteristics. The median year since the first global record of introduction in our dataset is 1954, reflecting a relatively recent influx of species, especially compared to the median time for plant invasions in terrestrial systems which is often 1900 or earlier (e.g.,^16^,^24^,^25^,^26^,^27^). Although introduction history in the ocean seems more recent, this largely reflects a limited historical baseline of species inventories compared to terrestrial biotas^28^,^29^—a notion supported by the high number of species designated as cryptogenic in marine environs^30^. Despite the older introductions, many terrestrial plant studies have similarly shown a significant positive influence of time on range size^18^,^27^. Other taxa with more active dispersal may achieve equilibrium distributions quickly, especially when confined to a small region, such as birds in New Zealand, which showed no correlation between invasion time and current range size^31^.

That non-native species are largely not at equilibrium is one explanation for why other environmental and biological trait variables did not hold much explanatory power. If a species is not occupying its full niche space, areas with suitable combinations of habitat and environmental characteristics may still have an absence of the species due to dispersal limitation. However, failure to detect an effect of invader traits or recipient environment on range size may also result from other explanations. Variables we did not include in our model may have large influences on some species’ ranges, especially biotic interactions such as competition, predation, and mutualism. It is difficult, for example, to quantify recipient community diversity, or other proxies for the intensity of biotic interactions at all the invaded locations in our database, and poor descriptions of native ranges for many invaders preclude using donor environment characteristics. Both of these aspects have been implicated in predicting invasion success^32^ and may account for some of the unexplained variance in our models.

Also, it is possible that no single variable is universally important in predicting species’ range sizes^33^,^34^, and so the failure of factors to contribute to range prediction occurs because species respond idiosyncratically to these factors. However, we view this as unlikely, given the first order importance of certain variables, such as salinity and temperature, in affecting organisms’ physiology, defining their ecological niches, and thus helping to set species biogeographic ranges^10^,^35^,^36^. It also seems unlikely that the lack of environmental correlates of range size stems from a poor characterization of the range due to under-sampling, poor data quality, or slow time to publication. While some under-reporting of occurrence data undoubtedly exists, the geographic region and species selected for analyses are well studied, serving to constrain such biases, and we did find a strong correlation of range with time since invasion. If lags exist in the discovery and reporting of invaders from their actual time of invasion, as long as these lags are random or unbiased across species, this effect should not affect the slope of the relationship between time since invasion and range size but would only shift the relationship to the right (lower the intercept of the relationship).

The lack of a standard influence of climate-related variables like temperature in our analysis is noteworthy, but is not inconsistent with reports that warming temperatures have facilitated invasions in some locations^37^ or resulted in range expansions of native species (e.g.,^38^,^39^). Even if a species’ range is expanded in one direction as a result of climate change, the total range size may still be a fraction of the potential range. Furthermore, we might expect climate change to have a minimal effect on total range size because expansions in one direction along a coastline may be balanced by contractions in another. In general, we urge caution in interpreting net climatic effects (or the lack thereof) for invasive species with potential ranges that are currently undersaturated.

In conclusion, the importance of the length of time since first global introduction implies that many invaders are still spreading, which increases the difficulty in detecting key trait and environmental controls of biological invasions from broad scale correlative analyses. Time since first global introduction is an appealing predictive variable because it is discrete, and relatively easy to quantify. However, unlike habitat and trait variables, it does not help to build a preventative model of spread since it is a variable that is quantifiable only post-invasion. Because the footprint of existing non-native species is still expanding, any consideration of their potential impact (*sensu 9*) is likely underestimated. Our findings further suggest that the current best general strategy to preemptively protect areas from invasion is to identify vulnerable areas based on vector inputs as opposed to site and species characteristics.

## Methods

### Choosing species for inclusion

We focused on marine benthic invertebrate species that are non-native to either Australia, New Zealand, or the United States because these countries have the most complete data available on distributional range for non-native species. We accumulated target species by first compiling a comprehensive list of introduced species from previously assembled, publicly available national port surveys and non-native species databases in these countries. For Australia we used the Australian port surveys’ list of known exotic species in Australian waters (summarized in^40^), the Australian National Consultative Committee on Introduced Marine Pest Emergencies (CCIMPE) trigger list species. For New Zealand we used the New Zealand Port Biological Baseline Survey list of introduced species (http://www.marinebiosecurity.org.nz/programme-information-port-survey/). For the United States, sources for our initial species list were the United States Geological Survey Nonindigenous Aquatic Species database (http://nas.er.usgs.gov/), the United States National Exotic Marine and Estuarine Species Information System (http://invasions.si.edu/nemesis/index.html), and the United States NOAA Technical Memorandum NOS NCCOS 77 (http://coastalscience.noaa.gov/documents/techmemo77.pdf).

We excluded most aquaculture species, as these species have been intentionally introduced and nurtured to thrive in their new environments, and often specific steps are taken in association with their introduction to either prevent or promote spread. We also excluded brackish species and species known to comprise a cryptic species complex, i.e., more than one species (e.g*. Namanereis littoralis)*.

### Data collection

#### Literature search: Compilation of species and their traits

First, we checked species taxonomies against the World Register of Marine Species (WoRMS, http://www.marinespecies.org/index.php), and acquired the accepted taxonomic name and associated synonyms. Then, for each species, we collected life history trait and invasion history data. Relevant life history traits included species’ temperature and salinity tolerances, depth range, larval duration, development type (planktonic or non-planktonic), maximum body size, habitat use (infaunal or epifaunal), and mobility (mobile or sessile). We also categorized species by phylum. We began our data gathering by mining the above databases, plus previously compiled species fact sheets from Australian and New Zealand governments (http://www.daff.gov.au/__data/assets/pdf_file/0008/2059154/species-biofouling-risk-assessment.pdf; NIWA pest sheets from G. Inglis), as well as fact sheets from the Bishop Museum and University of Hawaii, (http://www2.bishopmuseum.org/HBS/invertguide/species_pdf/guide.pdf). All information from fact sheets was double-checked with the referenced primary sources. We also extracted data from the following non-indigenous species online databases (searching for each species under all its synonyms): 1) The National Exotic Marine and Estuarine Species Information System (NEMESIS, http://invasions.si.edu/nemesis/index.html), 2) the National Introduced Marine Pest Information System (NIMPIS, http://adl.brs.gov.au/marinepests/index.cfm?fa=main.about), 3) the Global Invasive Species Database (http://www.issg.org/database/welcome/), 4) the Exotics Guide: Non-native Marine Species of the North American Pacific Coast (http://www.exoticsguide.org/), 5) the Invasive Species Compendium (http://www.cabi.org/isc/default.aspx?site=144&page=4066), 6) the European Network on Invasive Alien Species (NOBANIS, http://www.nobanis.org/), 7) Delivering Alien Invasive Species Inventories for Europe (DAISIE, http://www.europe-aliens.org/default.do), 8) the Marine Life Information Network (MarLIN, http://www.marlin.ac.uk/), and 9) the United States Geological Survey Nonindigenous Aquatic Species (NAS, http://nas.er.usgs.gov/).

To fill in missing data we then performed targeted literature searches of the gray literature and peer-reviewed journals in Web of Science and Google Scholar. Two life history traits—minimum duration of larval stage and development type—still had data missing after these literature searches. For these species we made reasonable assumptions for some life history traits based on species taxonomic group. For example, in the absence of a source, we assumed colonial ascidians to be lecithotrophic (yolk-feeding), with minimum larval durations of less than 24 hours. We kept data with added assumptions separate from data with concrete sources so that we could ascertain if inclusion of the assumptions differentially affected the model outcome. Several life history traits (e.g., larval temperature and salinity tolerance, larval duration, age and size at maturity, depth range) were still missing from more than 25% of the species and so they were no longer considered in the model construction. The final trait variables that had full representation in the dataset were: maximum body size, adult mobility (sessile or mobile), adult habitat (epifaunal or infaunal), and larval development type (planktonic or non-planktonic). Overall, the final dataset contained 138 species.

From the literature we also extracted for each species its time since the first record of introduction anywhere in the world.

#### Quantifying distribution data

We used species occurrence data from the Global Biodiversity Information Facility database (GBIF, http://www.gbif.org/) to quantify species ranges. Specifically we calculated the total length of coastline occupied by a species in its non-native range.

Ranges were determined for each non-native species by first downloading from GBIF exact latitudinal and longitudinal coordinates for each point of the species’ occurrence and then partitioning non-native from native occurrences using the previously described literature search. We then introduced the geographic coordinates for the non-native occurrences of each species into Google Earth, and used the measuring tool to measure ranges (in km), spanning occurrences, along the coastline of each ocean basin in both northern and southern hemispheres. That is, separate range distributions were calculated for the east and west coastlines of the Atlantic and Pacific Oceans in both the Northern and Southern Hemispheres. Where multiple points of occurrence occurred on large islands (e.g. Tasmania), ranges were measured around the coastline in the same manner as continents; occurrences on oceanic archipelagos >1000km from a continental coastline were treated as a separate distribution and ranges were measured as a straight line through the cluster of occurrences. All single, isolated points of occurrence were given a common distance measurement of 50 km. To prevent overestimation of ranges, if two neighboring points of occurrence on a coastline were >1500 km apart, then the range was not considered continuous through this stretch and the ranges on either side of those points were tabulated as disparate. The distances of ranges for all disparate segments along each coastline (and oceanic islands) were then summed across all coasts to give a total range size in km.

Any single, isolated points of occurrence in GBIF that had no corroborating documentation within the literature were ignored. (Such instances were few, and their inclusion would have only added 50 km to the total calculated range). We also eliminated coordinates if they clearly exhibited human error, such as points with zero values for both latitude and longitude, or if coordinates were located inland (e.g. as is sometimes the case for museum specimens).

For 25 known non-native species without GBIF occurrences, we assigned coordinates to the range distributions extracted during our extensive literature search. For most of these species, exact latitudinal coordinates were given, but in a handful of cases only general descriptions were given (e.g. “coast of California”), in which case we assigned coordinates by recording a geographic coordinate every 200 km along the coastline of the reported locale (e.g., every 200 km along the coast of California). To test the concordance of the GBIF and literature-based data gathering approaches, we applied the literature-based method to ten randomly chosen species with available GBIF coordinates to calculate how similar the non-native ranges were between these two methods. Ranges of the two methods were generally congruent, although species with available GBIF occurrence data tended to slightly underestimate the literature-extracted geographic ranges. We organized the range distribution data to allow for analysis of species collected with GBIF methodology both separately and combined with the species extracted with literature-based methods. Finding no substantial differences between the pooled data and the GBIF data alone, we used the pooled dataset for formal analyses.

#### Physical data

We collected seasonal and annual oceanographic data for each species in its non-native geographic ranges using the same coordinates of species occurrence points we extracted from GBIF (or the literature for those 25 species). We extracted oceanographic parameters—current speed, temperature, and salinity—for each species occurrence point from the closest one-degree by one-degree boxes included in both the World Ocean Atlas 2009 (http://www.nodc.noaa.gov/OC5/WOA09/pr_woa09.html) and the Drifter Data Assembly Center (http://www.aoml.noaa.gov/phod/dac/dacdata.php). This is the highest resolution global data set with coastal coverage that includes ocean currents. We calculated the mean, standard deviation, minimum, and maximum for each parameter across all occurrences for all seasons (defined as standard oceanographic seasons for northern and southern hemispheres), as well as for the year in total. An annual range in temperature and salinity was also calculated.

### Analyses

We used a model information-theoretic framework to test the relative influence of time since introduction, biological characteristics of species, and physical habitat properties on non-native range distributions. Residuals of the data were nearly normal, and so they did not require transformation. We fit multiple linear regression models to the measured cumulative range size of each species and selected the minimally adequate models with corrected Akaike’s Information Criterion (AIC_c_). We estimated the standardized beta coefficients of all variables (including categorical ones) to enable comparisons of relative importance among the predictor variables.

Predictor variables included time since introduction, habitat (epifaunal vs infaunal), mobility (sessile vs mobile), maximum body size, development type (planktonic vs non-planktonic), mean current speed in spring, annual current variability, mean spring temperature, annual standard deviation in temperature, annual mean salinity, and annual standard deviation in salinity. Minimum larval duration, a trait that was only available for a small subset of the species, was also explored but showed extremely little effect, so was removed to increase the overall degrees of freedom in the model by having a more complete dataset. Instead, life history attributes like development type were considered a categorical proxy for larval duration.

We pared down the large list of physical variables by generating a covariance matrix of seasonal and annual temperatures, and then again for salinity. Many variables were highly correlated (R > 0.9), and among such clusters we selected one representative variable among those where R > 0.7 for entry into the final model competitions. Mostly, this meant using yearly averages and standard deviations. The only exceptions were for mean current speed and temperature for which we used spring values since this is the peak period of spawning for many species with broadcast larvae^41^ and thus a time when larvae in the water column, and thus spread rates of populations, can be most influenced by these variables^42^.

To identify a minimally adequate model, we first fit linear regressions with all possible combinations of independent variables. A null (intercept only) model was also compared. Next, we selected the model with the lowest AIC_c_ and calculated ΔAIC_c_ for each model, which indicates the difference in model parsimony as explained by AIC_c_ relative to the best model; lower ΔAIC_c_ values indicate higher support for a model. Because other models may be nearly as good (AIC_c_ nearly as low), we also calculated Akaike weights (w_i_) across the four best models for each number of variables up to seven^43^. Finally, for each variable included in the models we calculated its Relative Variable Importance (RVI), which is the sum of Akaike weights across all models that included that particular variable. Because of the large number of models, we conducted these RVI calculations on a subset composed of the best 40 models (lowest AIC_c_). AIC_c_ is a conservative version of AIC and often used when sample sizes are small. Although we were not overly concerned with small sample sizes, AIC_c_ converges on AIC as sample size increases and is considered a more conservative metric^44^.

To determine the robustness of results we also analyzed data with conditional random forest^45^ and boosted regression trees^46^. Tree-based algorithms have different assumptions to linear regression, and consistency between predictions of various methods can be used to gauge confidence in results. Random forest and boosted regression trees were done using the party^45^ and dismo packages in R v. 3.1.1, respectively.

Consistent with linear regression, both conditional random forest and boosted regression trees found ‘time since introduction’ to be the overwhelmingly strongest predictor (Fig. S1, Supp Materials 3). It was considered over 7 times more important than any other variable by conditional forest, and approximately 3 times more important by boosted regression trees. Both algorithms rank mean spring temperature and mean annual salinity the second and third most important variables respectively, but both with much lower importance than time since introduction.

## Additional Information

**How to cite this article**: Byers, J. E. *et al*. Invasion Expansion: Time since introduction best predicts global ranges of marine invaders. *Sci. Rep*. **5**, 12436; doi: 10.1038/srep12436 (2015).

## Supplementary Material

Supplementary Information

## Figures and Tables

**Figure 1:**
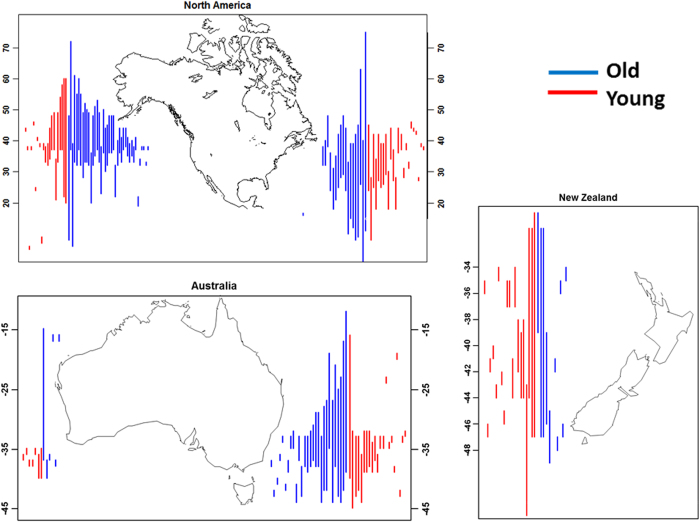
Ranges, distribution and invasion age of species in our study. Waterfall plot of the coastal ranges of non-native marine invertebrate species along the focal coastlines of this study shown as the latitudinal bands of species’ distributions. Some species may appear in more than one panel if present on more than one coast. Blue represents species whose first record of introduction anywhere in the world is before 1954 (i.e. the median time in our dataset, “Old”); red represents species whose first record of introduction anywhere in the world is between 1954–2012 (“Young”). New Zealand plot does not differentiate between east and west coasts. Figure was created in R 3.1.

**Figure 2:**
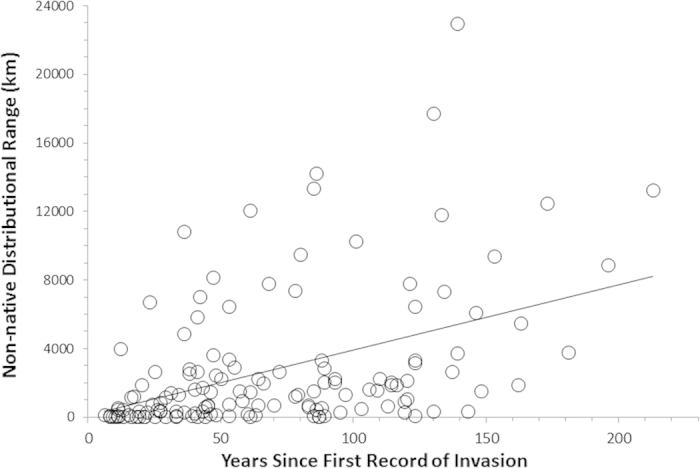
Relationship between a species’ time since first record of introduction anywhere and the length of coastline it occupies in its non-native range. Time since invasion is the single-most influential variable on range, explaining 20% of the variability. Range = 37.8 x Years since first record of global invasion + 138 (R^2^ = 0.20, P < 0.0001).

**Table 1:** Models explaining the coastal range (in km) occupied by non-native marine invertebrates.

Model ID	# variables	R^2^	AIC_c_	ΔAIC_c_	w_i_	Time since Introduction	Independent Variables									
							Biological				Physical					
							Habitat	Mobility	Max body size	Developmental type	Current speed (spring)	Current variability	Temperature mean (spring)	Temperature standard dev	Salinity mean	Salinity standard dev
Null	0	—	2643.4	38.9	4.7 × 10^−10^											
**A**	**4**	**0.29**	**2604.5**	**0.0**	**0.134**	***0.42***	–***0.19***		***0.15***						***−0.20***	
B	5	0.30	2605.3	0.8	0.090	*0.42*	–*0.19*		*0.15*				−0.08		–0.16	
C	5	0.30	2605.6	1.1	0.078	*0.41*	–*0.18*	–0.08	0.14						–*0.21*	
D	6	0.31	2605.7	1.2	0.073	*0.40*	–*0.20*		*0.15*			0.13	–0.17		–0.17	
E	4	0.29	2605.9	1.4	0.068	*0.45*	–*0.20*		*0.15*				–*0.15*			
F	5	0.30	2606.1	1.6	0.061	*0.42*	–*0.19*		0.14	–0.07					–*0.20*	
G	5	0.30	2606.4	1.9	0.052	*0.41*	–*0.20*		*0.16*			0.05			–*0.22*	
H	6	0.31	2606.6	2.1	0.047	*0.41*	–*0.18*	–0.08	0.13				–0.07		–*0.17*	
I	7	0.32	2606.7	2.2	0.045	*0.39*	–*0.18*	–0.09	0.13			0.14	–0.16		–*0.19*	
J	3	0.27	2606.8	2.3	0.042	*0.41*	–*0.18*								–*0.21*	
K	6	0.31	2606.9	2.4	0.040	*0.42*	–*0.19*		0.13	–0.07			–0.08		–0.16	
L	4	0.28	2606.9	2.4	0.040	*0.40*	–*0.16*	–0.11							–*0.23*	
M	7	0.32	2607.0	2.4	0.040	*0.40*	–*0.19*		0.13	–0.08		0.14	–0.17		–*0.18*	
N	4	0.28	2607.2	2.7	0.035	*0.41*	–*0.18*						–0.10		–0.16	
O	7	0.32	2607.3	2.8	0.033	*0.39*	–*0.20*		0.15		–0.10	0.22	–0.19		–0.17	
P	6	0.30	2607.3	2.8	0.033	*0.40*	–*0.18*	–0.09	0.14			0.06			–*0.24*	
Q	7	0.31	2608.0	3.4	0.024	*0.40*	–0.20		*0.15*			0.13	–0.17		–0.16	0.02
R	3	0.26	2608.0	3.5	0.023	*0.45*	–*0.19*						–*0.17*			
S	3	0.26	2608.9	4.4	0.015	*0.46*	–*0.20*		*0.18*							
T	3	0.26	2609.2	4.6	0.013	*0.39*		0.15							–*0.23*	
U	2	0.24	2610.7	6.2	0.006	*0.40*									–*0.22*	
V	2	0.23	2611.8	7.3	0.004	*0.44*							–*0.18*			
W	2	0.23	2612.2	7.7	0.003	*0.45*	–*0.19*									
X	2	0.22	2613.6	9.1	0.001	*0.45*			*0.16*							
Y	1	0.20	2616.0	11.4	0.0004	*0.44*										
Z	1	0.09	2632.9	28.4	9.1 × 10^–8^										–*0.30*	
AA	1	0.05	2638.7	34.2	5.1 × 10^–9^								–*0.19*			
BB	1	0.02	2642.2	37.7	8.9 × 10^−10^		–0.16									
					**RVI**	**1.00**	**1.00**	**0.26**	**0.83**	**0.18**	**0.08**	**0.28**	**0.55**	**0.08**	**0.84**	**0.12**

The four best fitting models are shown for each number of variables up to seven; with eight or more variables, models fit poorly (ΔAIC_c_ value > 3.7). The standardized beta coefficients associated with each independent variable are shown for each model (italics represent coefficients significant at P < 0.05 level). The best, most parsimonious model with the lowest Akaike information criterion (AIC_c_) value is shown in bold. A null (intercept only) model is also compared. ΔAIC_c_ indicates the difference in model parsimony as explained by AIC_c_ relative to the best model; lower ΔAIC_c_ values indicate higher support for a model. Values of R^2^ and Akaike weight (w_i_) for each model are also shown. Akaike weights were calculated across the models shown in table. RVI (relative variable importance) is the sum of the weights (w_i_) of all models containing a particular parameter and were calculated across the best 40 models (lowest AIC_c_). Habitat represents whether species is epifaunal or infaunal; mobility is sessile or mobile. Beta coefficients indicate that distributional extent decreases if habitat is epifaunal, and decreases for species that are mobile as adults.

## References

[b1] Reichard S. H. & Hamilton C. W. Predicting invasions of woody plants introduced into North America. Conservation Biology 11, 193–203 (1997).

[b2] Byers J. E. . Directing research to reduce the impacts of nonindigenous species. Conservation Biology 16, 630–640 (2002).

[b3] Drake J. A. . Ecology of biological invasions: a global perspective. (John Wiley and Sons, 1989).

[b4] Williamson M. H. & Fitter A. The characters of successful invaders. Biological Conservation 78, 163–170 (1996).

[b5] Heger T. & Trepl L. Predicting biological invasions. Biological Invasions 5, 313–321 (2003).

[b6] Kulhanek S. A., Ricciardi A. & Leung B. Is invasion history a useful tool for predicting the impacts of the world’s worst aquatic invasive species? Ecol. Appl. 21, 189–202 (2011) 21516897 10.1890/09-1452.1

[b7] Gaither M. R., Bowen B. W. & Toonen R. J. Population structure in the native range predicts the spread of introduced marine species. P. Roy. Soc. B-Biol. Sci. 280, 20130409 (2013). 10.1098/rspb.2013.0409 PMC3652461 23595272

[b8] Dick J. T. A. . Advancing impact prediction and hypothesis testing in invasion ecology using a comparative functional response approach. Biological Invasions 16, 735–753 (2014).

[b9] Parker I. . Impact: toward a framework for understanding the ecological effects of invaders. Biological Invasions 1, 3–19 (1999).

[b10] Gaston K. J. The structure and dynamics of geographic ranges. (Oxford University Press, 2003).

[b11] Emlet R. B. Developmental mode and species geographic range in regular sea urchins (Echinodermata, Echinoidea). Evolution 49, 476–489 (1995). 10.1111/j.1558-5646.1995.tb02280.x 28565076

[b12] Maliska M. E., Pennell M. W. & Swalla B. J. Developmental mode influences diversification in ascidians. Biol. Letters 9, doi: 10.1098/rsbl.2013.0068 (2013). 10.1098/rsbl.2013.0068 PMC3645039 23554280

[b13] Kohn A. J. Egg size, life history, and tropical marine gastropod biogeography. Am. Malacol. Bull. 30, 163–174 (2012).

[b14] Roy K., Jablonski D. & Valentine J. W. Climate change, species range limits and body size in marine bivalves. Ecology Letters 4, 366–370 (2001).

[b15] Roy K., Jablonski D. & Valentine J. W. Body size and invasion success in marine bivalves. Ecology Letters 5, 163–167 (2002).

[b16] Scott J. K. & Panetta F. D. Predicting the Australian weed status of southern African plants. Journal of Biogeography 20, 87–93 (1993).

[b17] Burlakova L. E., Karatayev A. Y. & Padilla D. K. The impact of *Dreissena polymorpha* (Pallas) invasion on unionid bivalves. Int. Rev. Hydrobiol. 85, 529–541 (2000).

[b18] Pyšek P., Krivanek M. & Jarosik V. Planting intensity, residence time, and species traits determine invasion success of alien woody species. Ecology 90, 2734–2744 (2009). 19886483 10.1890/08-0857.1

[b19] Moore K. A. & Elmendorf S. C. Propagule vs. niche limitation: untangling the mechanisms behind plant species’ distributions. Ecology Letters 9, 797–804 (2006). 16796569 10.1111/j.1461-0248.2006.00923.x

[b20] Clark G. F. & Johnston E. L. Temporal change in the diversity-invasibility relationship in the presence of a disturbance regime. Ecology Letters 14, 52–57 (2011). 21070561 10.1111/j.1461-0248.2010.01550.x

[b21] Ruiz G. M. & Carlton J. T. Invasive Species: Vectors And Management Strategies. (Island Press, 2003).

[b22] Freeman A. S. & Byers J. E. Divergent induced responses to an invasive predator in marine mussel populations. Science 313, 831–833 (2006). 16902136 10.1126/science.1125485

[b23] La Sorte F. A. & Pyšek P. Extra-regional residence time as a correlate of plant invasiveness: European archaeophytes in North America. Ecology 90, 2589–2597 (2009). 19769136 10.1890/08-1528.1

[b24] Rejmanek M. Invasive plants: approaches and predictions. Austral. Ecol. 25, 497–506 (2000).

[b25] Pyšek P., Sadlo J., Mandak B. & Jarosik V. Czech alien flora and the historical pattern of its formation: what came first to Central Europe? Oecologia 135, 122–130 (2003). 12647111 10.1007/s00442-002-1170-7

[b26] Hamilton M. A. . Life-history correlates of plant invasiveness at regional and continental scales. Ecology Letters 8, 1066–1074 (2005).

[b27] Huang Q. Q. . Determinants of the geographical extent of invasive plants in China: effects of biogeographical origin, life cycle and time since introduction. Biodivers. Conserv. 19, 1251–1259 (2010).

[b28] Grosholz E. Ecological and evolutionary consequences of coastal invasions. Trends Ecol. Evol. 17, 22–27 (2002).

[b29] Mead A., Carlton J. T., Griffiths C. L. & Rius M. Revealing the scale of marine bioinvasions in developing regions: a South African re-assessment. Biological Invasions 13, 1991–2008 (2011).

[b30] Carlton J. T. Biological invasions and cryptogenic species. Ecology 77, 1653–1655 (1996).

[b31] Duncan R. P., Blackburn T. M. & Veltman C. J. Determinants of geographical range sizes: a test using introduced New Zealand birds. Journal of Animal Ecology 68, 963–975 (1999).

[b32] Fridley J. D. & Sax D. F. The imbalance of nature: revisiting a Darwinian framework for invasion biology. Global Ecol. Biogeogr. 23, 1157–1166 (2014).

[b33] Lester S. E., Ruttenberg B. I., Gaines S. D. & Kinlan B. P. The relationship between dispersal ability and geographic range size. Ecology Letters 10, 745–758 (2007). 17594430 10.1111/j.1461-0248.2007.01070.x

[b34] Hayes K. R. & Barry S. C. Are there any consistent predictors of invasion success? Biological Invasions 10, 483–506 (2008).

[b35] O’Connor M. I. . Temperature control of larval dispersal and the implications for marine ecology, evolution, and conservation. P. Natl. Acad. Sci. USA 104, 1266–1271 (2007). 10.1073/pnas.0603422104 PMC1764863 17213327

[b36] Marshall D. J., Krug P. J., Kupriyanova E. K., Byrne M. & Emlet R. B. The Biogeography of Marine Invertebrate Life Histories. Annual Review of Ecology, Evolution, and Systematics. 43, 97–114 (2012).

[b37] Stachowicz J. J., Terwin J. R., Whitlatch R. B. & Osman R. W. Linking climate change and biological invasions: Ocean warming facilitates nonindigenous species invasions. P. Natl. Acad. Sci. USA 99, 15497–15500, (2002). 10.1073/pnas.242437499 PMC137745 12422019

[b38] Booth D. J., Bond N. & Macreadie P. Detecting range shifts among Australian fishes in response to climate change. Mar. Freshwater Res. 62, 1027–1042 (2011).

[b39] Verges A. . The tropicalization of temperate marine ecosystems: climate-mediated changes in herbivory and community phase shifts. P. Roy. Soc. B-Biol. Sci. 281, Artn 20140846 (2014). 10.1098/rspb.2014.0846 PMC4100510 25009065

[b40] Bishop M. J. & Hutchings P. A. How useful are port surveys focused on target pest identification for exotic species management? Mar. Pollut. Bull. 62, 36–42 (2011). 20934194 10.1016/j.marpolbul.2010.09.014

[b41] Reitzel A. M., Miner B. G. & McEdward L. R. Relationships between spawning date and larval development time for benthic marine invertebrates: a modeling approach. Marine Ecology-Progress Series 280, 13–23 (2004).

[b42] Byers J. E. & Pringle J. M. Going against the flow: retention, range limits and invasions in advective environments. Mar. Ecol. Prog. Ser. 313, 27–41 (2006).

[b43] Burnham K. P. & Anderson D. R. Model Selection and Multimodel Inference: A Practical Information-Theoretic Approach. 2 edn, (Springer, 2002).

[b44] Burnham K. P., Anderson D. R. & Huyvaert K. P. AIC model selection and multimodel inference in behavioral ecology: some background, observations, and comparisons. Behav. Ecol. Sociobiol. 65, 23–35 (2011).

[b45] Strobl C., Boulesteix A., Kneib T., Augustin T. & Zeileis A. Conditional Variable Importance for Random Forests. BMC Bioinformatics 9, 307 (2008). 18620558 10.1186/1471-2105-9-307 PMC2491635

[b46] Elith J., Leathwick J. R. & Hastie T. A working guide to boosted regression trees. Journal of Animal Ecology 77, 802–813 (2008). 18397250 10.1111/j.1365-2656.2008.01390.x

