# Supplementary material for: Invasion Expansion: Time since introduction best predicts global ranges of marine invaders
Source: Sci Rep. 2015 Jul 31;5:12436. doi: 10.1038/srep12436 (PMC4521186; doi:10.1038/srep12436)
Supplement: Supplementary Information [file srep12436-s1.pdf]

Supplementary Information for:

Invasion Expansion: Time since introduction best predicts global ranges of marine invaders

James E. Byers[1*], Rachel S. Smith[1,3], James M. Pringle[2], Graeme F. Clark[3], Paul E. Gribben[4], Chad L. Hewitt[5], Graeme J. Inglis[6], Emma L. Johnston[3], Gregory M. Ruiz[7], John J. Stachowicz[8], Melanie J. Bishop[9]

Affiliations:

[1]Odum School of Ecology, University of Georgia, Athens, GA 30602 USA.

[2]142 Morse Hall, Ocean Process Analysis Laboratory, 8 College Rd, UNH, Durham NH 03857 USA.

[3]Evolution & Ecology Research Centre, School of Biological, Earth, and Environmental Sciences, University of New South Wales, Sydney 2052 NSW Australia

[4]Centre of Marine Bio-innovation, School of Biological, Earth and Environmental Science, University of New South Wales, Sydney 2052 NSW Australia

[5]School of Science, University of Waikato, Hamilton 3240, New Zealand.

[6]National Institute of Water and Atmospheric Research, 10 Kyle Street, Riccarton, Christchurch 8011, New Zealand

[7]Smithsonian Environmental Research Center, 647 Contees Wharf Road, Edgewater, MD 21037 USA.

[8]Department of Evolution and Ecology, University of California, Davis, CA 95616 USA

[9]Department of Biological Sciences, Macquarie University, North Ryde, NSW 2109 Australia.

*Correspondence to: jebyers@uga.edu

Supplemental Materials 1:

Our database of species and their range and trait characteristics.

Species	Phylum	Mobility	Habitat	Development type	Max body size (mm)	Time since introduction (years prior to 2012)	Total non-native Range (km)
Alitta succinea	Annelid	Sessile	Infauna	P	190	153	9406
Amphibalanus amphitrite	Arthropod	Sessile	Epifauna	P	30	130	17731
Amphibalanus eburneus	Arthropod	Sessile	Epifauna	P	25	97	1334
Amphibalanus reticulatus	Arthropod	Sessile	Epifauna	P	18	57	1504
Amphibalanus subalbidus	Arthropod	Sessile	Epifauna	P	20	24	749
Amphisbetia maplestonei	Hydrozoan	Sessile	Epifauna	P	50	8	100
Ampithoe longimana	Arthropod	Mobile	Epifauna	NP	40	64	712
Ampithoe valida	Arthropod	Mobile	Epifauna	NP	15	72	2659
Arcuatula senhousia	Mollusc	Sessile	Infauna	P	35	89	2852
Ascidiella aspersa	Chordata	Sessile	Epifauna	P	130	114	1892
Assiminea parasitologica	Mollusc	Mobile	Epifauna	P	5.8	6	165
Asterias amurensis	Echinoderm	Mobile	Epifauna	P	500	27	882
Astrostole scabra	Echinoderm	Mobile	Epifauna	P	400	44	50
Balanus glandula	Arthropod	Sessile	Epifauna	P	23.8	39	100
Balanus trigonus	Arthropod	Sessile	Epifauna	P	25	146	6127
Barantolla lepte	Annelid	Mobile	Infauna	P*	60	15	150
Batillaria attramentaria	Mollusc	Mobile	Epifauna	NP	54	93	2031
Blackfordia virginica	Hydrozoan	Sessile	Epifauna	P	22	109	1564
Boccardia proboscidea	Annelid	Sessile	Epifauna	P	35	38	2833
Boccardiella ligerica	Annelid	Sessile	Epifauna	P	26	78	7413
Boonea bisuturalis	Mollusc	Mobile	Epifauna	P	5.8	36	304
Botrylloides diegensis	Chordata	Sessile	Epifauna	P	150	41	50
Botrylloides violaceus	Chordata	Sessile	Epifauna	P	1500	47	8138
Botryllus planus	Chordata	Sessile	Epifauna	P	40	44	610
Brachidontes pharaonis	Mollusc	Sessile	Epifauna	P	40	137	2683

Bugula flabellata	Bryozoan	Sessile	Epifauna	P	40	88	3318
Bugula stolonifera	Bryozoan	Sessile	Epifauna	P	40	53	6476
Cancer gibbulosus	Arthropod	Mobile	Epifauna	P	20	8	50
Cancer novaezelandiae	Arthropod	Mobile	Epifauna	P	160	83	627
Caprella mutica	Arthropod	Mobile	Epifauna	NP	35	36	4857
Carcinus maenas	Arthropod	Mobile	Epifauna	P	100	196	8883
Celleporaria nodulosa	Bryozoan	Sessile	Epifauna	P	140	11	569
Charybdis hellerii	Arthropod	Mobile	Epifauna	P	79	89	2061
Charybdis japonica	Arthropod	Mobile	Epifauna	P	120	13	50
Chiton glaucus	Mollusc	Mobile	Epifauna	P	55	103	505
Chondropsis topsenti	Sponge	Sessile	Epifauna	P	200	25	50
Chthamalus proteus	Arthropod	Sessile	Epifauna	P	10	40	250
Ciona savignyi	Chordata	Sessile	Epifauna	P	150	110	2224
Cirolani harfordi	Arthropod	Mobile	Epifauna	NP	22	41	2673
Clytia kincaidi	Hydrozoan	Sessile	Epifauna	P	3	78	1202
Conopeum seurati	Bryozoan	Sessile	Epifauna	P	20	50	2267
Convoluta convoluta	Xenacoelomorpha	Mobile	Epifauna	NP	9	18	50
Corbula gibba	Mollusc	Sessile	Infauna	P	20	26	452
Cordylophora caspia	Hydrozoan	Sessile	Epifauna	P	100	213	13267
Crepidula convexa	Mollusc	Sessile	Epifauna	NP	20	43	1708
Crepidula fornicata	Mollusc	Sessile	Epifauna	P	58	133	11826
Crepidula plana	Mollusc	Sessile	Epifauna	P	43	148	1502
Cryptosula pallasiana	Bryozoan	Sessile	Epifauna	P	30	123	6446
Cuthona perca	Mollusc	Mobile	Epifauna	P*	16	46	200
Cyclicopora longipora	Bryozoan	Sessile	Epifauna	P	21	21	50
Cyrenoida floridana	Mollusc	Mobile	Epifauna	NP	19	61	50
Diadumene leucolena	Cnidaria	Sessile	Epifauna	P	38	34	1345
Diadumene lineata	Cnidaria	Sessile	Epifauna	P	40	121	7784
Didemnum psammatode	Chordata	Sessile	Epifauna	P	80	33	100
Ecteinascidia turbinata	Chordata	Sessile	Epifauna	P	140	53	84
Electra angulata	Bryozoan	Sessile	Epifauna	P	36	21	50

Eriocheir japonica	Arthropod	Mobile	Epifauna	P	100	16	50
Eriocheir sinensis	Arthropod	Mobile	Epifauna	P	100	101	10258
Euchone limnicola	Annelid	Sessile	Infauna	P*	12	29	1181
Eudendrium generale	Hydrozoan	Sessile	Epifauna	P	300	11	100
Eupleura sulcidentata	Mollusc	Mobile	Epifauna	P	24	17	1239
Eurylana arcuata	Arthropod	Mobile	Epifauna	NP	20	88	560
Gemma gemma	Mollusc	Sessile	Infauna	NP	5	114	2048
Geukensia demissa	Mollusc	Sessile	Epifauna	P	130	119	929
Glebocarcinus amphioetus	Arthropod	Mobile	Epifauna	P	41	79	1324
Godiva quadricolor	Mollusc	Mobile	Epifauna	P*	30	36	10815
Grandidierella japonica	Arthropod	Mobile	Epifauna	NP	13	47	3648
Grantessa intusarticulata	Sponge	Sessile	Epifauna	P	60	87	50
Halicarcinus innominatus	Arthropod	Mobile	Epifauna	NP	19	87	50
Hemigrapsus sanguineus	Arthropod	Mobile	Epifauna	P	42	25	2671
Hippoporina indica	Bryozoan	Sessile	Epifauna	P	50	12	4020
Hydroides ezoensis	Annelid	Sessile	Epifauna	P	45	40	1652
Ilyanassa obsoleta	Mollusc	Mobile	Epifauna	P	30	106	1626
Ischadium recurvum	Mollusc	Sessile	Epifauna	P*	50	143	338
Janolus hyalinus	Mollusc	Mobile	Epifauna	P*	30	27	349
Jassa marmorata	Arthropod	Mobile	Epifauna	NP	7	134	7333
Jassa staudei	Arthropod	Mobile	Epifauna	NP	11.4	9	50
Limaria orientalis	Mollusc	Sessile	Infauna	P	30	41	5822
Limnoria quadripunctata	Arthropod	Mobile	Epifauna	NP	4	163	5487
Littorina littorea	Mollusc	Mobile	Epifauna	P	52	173	12481
Littorina saxatilis	Mollusc	Mobile	Epifauna	NP	18	20	1882
Loxosomatoides laevis	Entoprocta	Sessile	Epifauna	P	0.98	19	50
Maoricolpus roseus	Mollusc	Sessile	Epifauna	P	90	93	2236
Megabalanus coccopoma	Arthropod	Sessile	Epifauna	P	50	162	1897
Megabalanus rosa	Arthropod	Sessile	Epifauna	P	41	60	191
Melampus floridanus	Mollusc	Mobile	Epifauna	P	7	119	301
Melita matilda	Arthropod	Mobile	Epifauna	NP	9	11	50

Membraniporopsis tubigera	Bryozoan	Sessile	Epifauna	P	60	16	1181
Microcosmus squamiger	Chordata	Sessile	Epifauna	P	50	95	300
Molgula manhattensis	Chordata	Sessile	Epifauna	P	70	68	7802
Mya arenaria	Mollusc	Sessile	Infauna	P	150	139	22985
Myosotella myosotis	Mollusc	Mobile	Epifauna	NP	8	181	3775
Mytilopsis sallei	Mollusc	Sessile	Epifauna	P	35	64	2237
Neilo australis	Mollusc	Sessile	Infauna	P*	53	48	150
Notomegabalanus algicola	Arthropod	Sessile	Epifauna	P	10	70	705
Obelia longissima	Hydrozoan	Sessile	Epifauna	P	350	85	13329
Okenia eolida	Mollusc	Mobile	Epifauna	P*	15	53	776
Paracerceis sculpta	Arthropod	Mobile	Epifauna	NP	8.5	45	711
Paracorophium brisbanensis	Arthropod	Mobile	Epifauna	NP	4.4	11	428
Paradella dianae	Arthropod	Mobile	Epifauna	NP	5.6	46	1455
Patiriella regularis	Echinoderm	Mobile	Epifauna	P	45	61	1501
Pennaria disticha	Hydrozoan	Sessile	Epifauna	P	300	85	1537
Perophora japonica	Chordata	Sessile	Epifauna	P	6	31	1400
Petrolisthes armatus	Arthropod	Mobile	Epifauna	P	14	19	341
Petrolisthes elongatus	Arthropod	Mobile	Epifauna	P	21	113	672
Philine orientalis	Mollusc	Mobile	Epifauna	P*	50	23	6716
Plumularia pulchella	Hydrozoan	Sessile	Epifauna	P	15	85	100
Polycera capensis	Mollusc	Mobile	Epifauna	P*	70	86	446
Potamocorbula amurensis	Mollusc	Sessile	Infauna	P	27.5	27	408
Pseudopolydora corniculata	Annelid	Sessile	Infauna	P	4	13	432
Pseudopolydora paucibranchiata	Annelid	Sessile	Infauna	P	15	61	12078
Pyromaia tuberculata	Arthropod	Mobile	Epifauna	P	26	38	2576
Rapana venosa	Mollusc	Mobile	Epifauna	P	180	66	2002
Rhithropanopeus harrisii	Arthropod	Mobile	Epifauna	P	30	139	3729
Sabella spallanzanii	Annelid	Sessile	Epifauna	P	500	48	2466
Schizoporella errata	Bryozoan	Sessile	Epifauna	P	400	53	3383
Schizoporella unicornis	Bryozoan	Sessile	Epifauna	P	40	86	14222
Sphaeroma quoyanum	Arthropod	Mobile	Epifauna	NP	15	120	2130

Sphaeroma serratum	Arthropod	Mobile	Epifauna	NP	10	33	340
Sphaeroma walkeri	Arthropod	Mobile	Epifauna	NP	10	120	1055
Stramonita haemastoma	Mollusc	Mobile	Epifauna	P	114	130	345
Styela clava	Chordata	Sessile	Epifauna	P	220	80	9482
Stylotella agminata	Sponge	Sessile	Epifauna	P	200	45	666
Symplectoscyphus indivisus	Hydrozoan	Sessile	Epifauna	P	20	10	50
Symplectoscyphus subdichotomus	Hydrozoan	Sessile	Epifauna	P	50	83	724
Symplegma reptans	Chordata	Sessile	Epifauna	P	300	22	270
Synidotea laevidorsalis	Arthropod	Mobile	Epifauna	NP	35	116	1879
Synthecium campylocarpum	Hydrozoan	Sessile	Epifauna	P	50	123	100
Synthecium subventricosum	Hydrozoan	Sessile	Epifauna	P	75	58	940
Tanais dulongii	Arthropod	Mobile	Epifauna	NP	7	43	324
Terebrasabella heterouncinata	Annelid	Sessile	Epifauna	P	5	33	50
Theora lubrica	Mollusc	Sessile	Infauna	P	15	55	2935
Tubificoides heterochaetus	Annelid	Mobile	Infauna	NP	9	42	7023
Urosalpinx cinerea	Mollusc	Mobile	Epifauna	NP	35	123	3156
Venerupis largillierti	Mollusc	Sessile	Infauna	P*	70	63	150
Vitrinella floridana	Mollusc	Mobile	Epifauna	P	2.1	29	61
Watersipora arcuata	Bryozoan	Sessile	Epifauna	P	300	123	3325
Zeacumantis subcarinatus	Mollusc	Mobile	Epifauna	NP	16	89	91

Notes: For development type P= planktonic, NP= non-planktonic, and * superscript indicates best guesses based on closely related species. For time since introduction blue font represents invasions older than the median year of introduction (1954), red is younger.

Supplemental Materials 2:

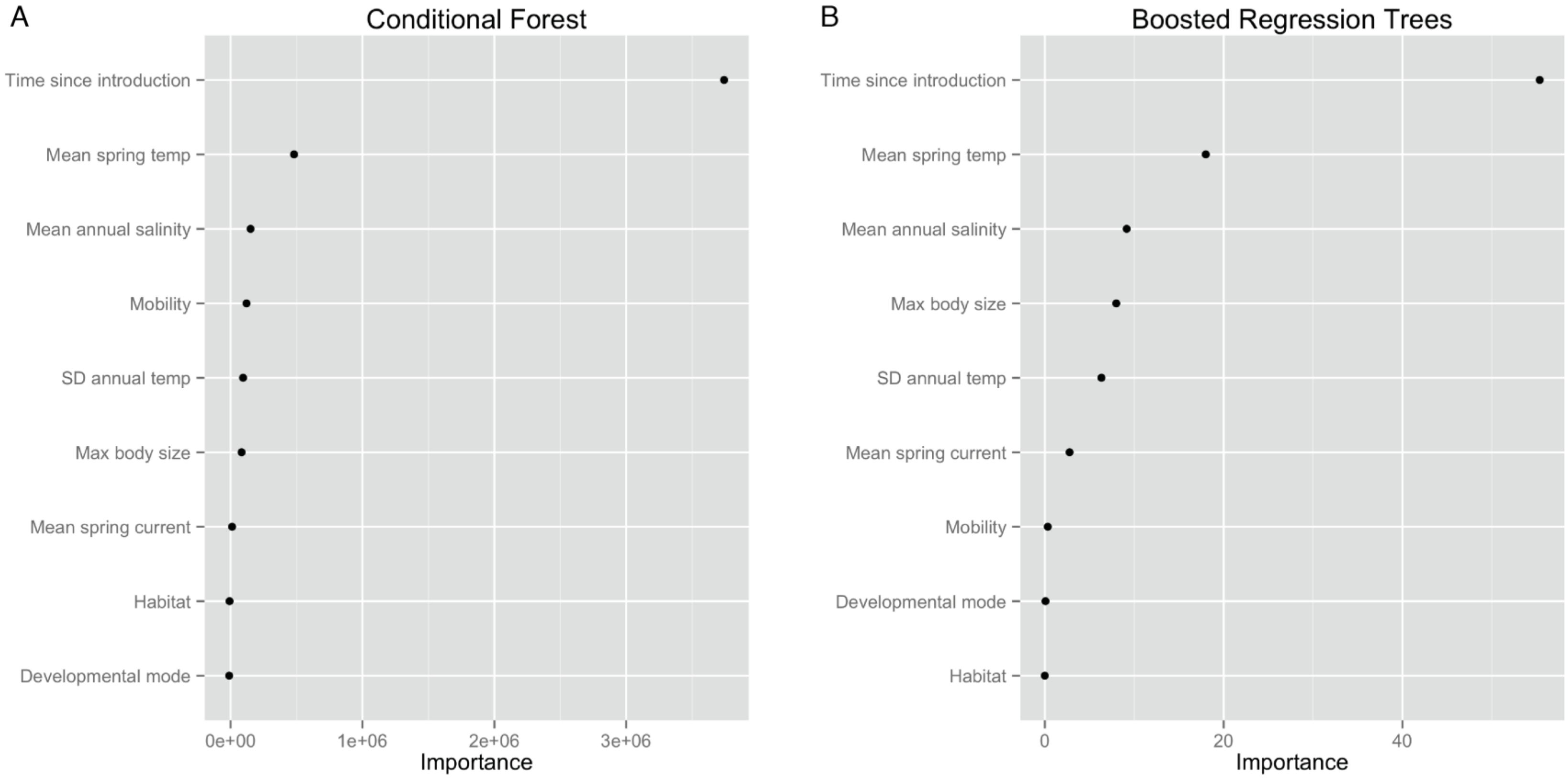

Figure S1. Relative importance of variables predicting non-native range (km of coastline) of introduced species, as determined by conditional random forest and boosted regression trees.
